# Two small-molecule inhibitors of *Toxoplasma gondii* proliferation *in vitro*

**DOI:** 10.3389/fcimb.2023.1145824

**Published:** 2023-04-03

**Authors:** Qian-qian Hua, Xue-jing Lin, Shi-peng Xiang, Li-ya Jiang, Jin-hao Cai, Jian-min Sun, Feng Tan, Ya-ni Mou

**Affiliations:** ^1^ Department of Clinical Laboratory, Affiliated Dongyang Hospital of Wenzhou Medical University, Dongyang, Zhejiang, China; ^2^ Department of Parasitology, School of Basic Medical Sciences, Wenzhou Medical University, Wenzhou, Zhejiang, China

**Keywords:** CGI-1746, JH-II-127, *Toxoplasma gondii*, infection, *in vitro*

## Abstract

**Background:**

Toxoplasmosis caused by *Toxoplasma gondii* is a globally distributed zoonosis. Most infections appear asymptomatic in immunocompetent individuals, but toxoplasmosis can be fatal in fetuses and immunocompromised adults. There is an urgent need to research and develop effective and low-toxicity anti-*T. gondii* drugs because of some defects in current clinical anti-*T. gondii* drugs, such as limited efficacy, serious side effects and drug resistance.

**Methods:**

In this study, 152 autophagy related compounds were evaluated as anti-*T. gondii* drugs. The activity of β-galactosidase assay based on luminescence was used to determine the inhibitory effect on parasite growth. At the same time, MTS assay was used to further detect the effects of compounds with over 60% inhibition rate on host cell viability. The invasion, intracellular proliferation, egress and gliding abilities of *T. gondii* were tested to assess the inhibitory effect of the chosen drugs on the distinct steps of the *T. gondii* lysis cycle.

**Results:**

The results showed that a total of 38 compounds inhibited parasite growth by more than 60%. After excluding the compounds affecting host cell activity, CGI-1746 and JH-II-127 were considered for drug reuse and further characterized. Both CGI-1746 and JH-II-127 inhibited tachyzoite growth by 60%, with IC_50_ values of 14.58 ± 1.52 and 5.88 ± 0.23 μM, respectively. TD_50_ values were 154.20 ± 20.15 and 76.39 ± 14.32 μM, respectively. Further research found that these two compounds significantly inhibited the intracellular proliferation of tachyzoites. Summarize the results, we demonstrated that CGI-1746 inhibited the invasion, egress and especially the gliding abilities of parasites, which is essential for the successful invasion of host cells, while JH-II-127 did not affect the invasion and gliding ability, but seriously damaged the morphology of mitochondria which may be related to the damage of mitochondrial electron transport chain.

**Discussion:**

Taken together, these findings suggest that both CGI-1746 and JH-II-127 could be potentially repurposed as anti-*T. gondii* drugs, lays the groundwork for future therapeutic strategies.

## Introduction

*Toxoplasma gondii* (*T. gondii*) is a globally endemic parasite that infects almost all warm-blooded animals. *T. gondii* is one of the most widespread parasites in the world, both geographically and in terms of the diversity of the host range it infects (Kochanowsky and Koshy, 2018). About one-third of the world’s population is infected with toxoplasmosis, which is a zoonotic disease, the prevalence of toxoplasmosis in livestock in China has reached 20%~50% (Li et al., 2015; Pan et al., 2017). *T. gondii* infection is mainly caused by eating raw meat, fruits, and vegetables infected with feline feces (Boyer et al., 2011; Mangiavacchi et al., 2016). *T. gondii* can also be transmitted vertically from mother to fetus, making the fetus a congenital carrier of *T. gondii*. The most serious harm caused by *T. gondii* is abortion, in addition, parasitemia and related tissue and organ damage can also be caused. Common symptoms include lymphadenopathy (Demar et al., 2007), central nervous system damage (Matta et al., 2021), pneumonia (Leal et al., 2007), myocarditis (Kirchhoff et al., 2004), toxoplasmosis chorioretinitis (McAuley, 2014) (vision loss, optic atrophy, cataract), endocrine disorders, *etc.* It is worth noting that toxoplasmosis has been an easily overlooked parasitic infection in the 110 years since its discovery by Nicolle & Manceaux. So far, no ideal therapeutic drug or vaccine for *T. gondii* infection exists. At present, the clinical anti- *T. gondii* drugs have been exposed with limited efficacy (Neville et al., 2015), serious side effects (Ben-Harari et al., 2017), drug resistance (Montazeri et al., 2018), and other defects, thus, there is an urgent need for safer and more effective clinical anti- *T. gondii* drugs.

The tachyzoites, bradyzoites and oocysts are the pathogenic or infectious stages to humans in nature, and tachyzoites form a marker of disease activity (Montoya and Liesenfeld, 2004). The combination of pyrimethamine and sulfadiazine (pyr-sulf) targeting the tachyzoite stage is the current gold standard for toxoplasmosis treatment, but the failure rate is still very high. Although other treatment options are available, including ethamide in combination with clindamycin, atovaquone, clarithromycin, or azithromycin, or with trimethoprim-sulfamethoxazole (TMP-SMX) or atovaquone alone, none is superior to pyr-sulf and none is effective against latent infections (Dunay et al., 2018). Therefore, we turned our attention to the most promising small-molecule drugs.

Small-molecule inhibitors are the primary agents for intracellular protein-targeted therapies. With high cellular permeability, small-molecule inhibitors can enter cells to block the activity of intracellular target proteins, interfering with downstream signaling pathways, and affecting the function of whole cells and tissues to provide specific therapeutic or preventive effects. In addition, the size of small-molecule inhibitors is usually in line with Lipinski’s rule of five (Lavanya et al., 2014; Wu et al., 2016), and the expected efficacy can be achieved by direct oral administration, with advantages of convenient use and long-lasting efficacy in clinical application. Autophagy is a protective mechanism of eukaryotic cells against stress and infection. Autophagosome and lysosome can digest and degrade abnormal protein aggregation and damaged organelles, which is an adaptive response of most cells in many aspects (Deretic et al., 2013; Cheng et al., 2022). In extracellular, *T. gondii* tachyzoites, autophagosomes are induced in response to amino acid starvation, but they can also be observed during normal intracellular development of the parasite (Besteiro et al., 2011). Because autophagy can play a protective or destructive role in different diseases, and even at different stages of the same disease. Many drugs and compounds that regulate autophagy are currently receiving considerable attention. For example, autophagy inducers such as trehalose (Sarkar et al., 2007), carbamazepine (Hidvegi et al., 2010); autophagy inhibitors such as Lys05 (Mcafee et al., 2012), and ATG7 inhibitors (Noda et al., 2011). Therefore, the development of other more specific and less toxic compounds to regulate the autophagy process can be effectively used for novel therapeutic strategies against *T. gondii*.

This study seeks to identify reusable small-molecule inhibitors of *T. gondii*. 152 compounds we have chosen are inhibitors against autophagic pathway, which were selected from 37,874 drugs at the National Drug Screening Center to identify those that inhibit *T. gondii* growth *in vitro*, further evaluating the effects of compounds with good anti-*T. gondii* activity and low host cell toxicity on tachyzoite division and proliferation *in vitro*. Among the 152 compounds screened in this study, two small-molecule inhibitors, CGI-1746 and JH-II-127 act as potential anti- *T. gondii* agents.

## Materials and methods

### Chemicals

The 152 small-molecule inhibitors associated with autophagy pathway (each stored as a 10 mM stock solution in DMSO) were obtained from the National Center for Drug Screening, China (http://www.screen.org.cn) and applied for preliminary screens against *T. gondii*. CGI-1746 (Cat *#*HY11999; MedChemExpress) and JH-II-127 (Cat *#*HY16936; MedChemExpress) were both obtained from MedChemExpress. These compounds were dissolved with dimethyl sulfoxide (DMSO, Sigma-Aldrich) into a 10 mM solution and stored at -80°C.

### Parasites and host cells

The *T. gondii* tachyzoites of the RH-2F strain, expressing luminescence-based β-galactosidase (β-Gal) (Seeber and Boothroyd, 1996) were maintained by repeat passage in monolayers of human foreskin fibroblasts (HFFs, ATCC, SCRC-1041) grown in Dulbecco’s modified Eagle’s medium (DMEM; Gibco, Invitrogen, Shanghai, China) supplemented with 10% (v/v) foetal bovine serum (FBS; Gibco, Invitrogen) and a cocktail of 1% (v/v) penicillin-streptomycin-glutamine (Cat #10378016, Gibco) at 37°C and 5% CO_2_.

### Preliminary screen

In the initial screening, the inhibitory effect on parasites growth was measured by β-Gal activity assays as described previously (Liu et al., 2020). Briefly, small molecular compounds were diluted with DMEM without phenol red to final concentrations of 10 μM and added to confluent monolayers of HFFs in 96-well plates. Then freshly collected RH-2F tachyzoites were added at a multiplicity of infection (MOI) of 0.2 (parasite/host cell ratio). Infected HFFs treated with 0.1% DMSO were used as the negative control, and 10 μM pyrimethamine, which is enough to inhibit *T. gondii* growth, was used as the positive control. After incubation for 72 h at 37°C and 5% CO_2_, chlorophenol red-β-d-galactopyranoside (CPRG, Sigma-Aldrich) with a final concentration of 100 μM was added to the medium and incubated for another 24h. At the end, the absorbance was detected at 570 nm. The number of parasites was derived from the standard curves generated in each plate.

To assess the cytotoxicity of these compounds that showed more than 60% parasite inhibition, a CellTiter 96R AQueous One Solution Cell Proliferation Assay system (Promega Corp, United States) with a 5-fold dilution was added to uninfected HFFs after 72 h of growth. The absorbance was recorded at 490 nm after the addition of reagent for 3 h. Compounds exhibiting low cytotoxicity (≥95% cell viability) were further assessed the drug efficacy.

### *In vitro* assessment of drug efficacy

To evaluate the inhibitory effects of compounds CGI-1746 and JH-I127 on parasites, β-Gal activity was experimented again. Both compounds were added to the first column of HFFs (~2000 cells/well) in a 96-well plate with an initial concentration of 200 μM and then diluted by serial 2-fold steps horizontally, leaving the final column drug-free. As a result, the final concentration of each well was 0, 0.39, 0.78, 1.56, 3.125, 6.25, 12.5, 25, 50, 100 and 200 μM, respectively. Two thousand of tachyzoites were then added per well in six of the eight rows. After 72 h incubation, the absorbance was detected at 570nm 24 h after addition of CPRG. Meanwhile, to measure the effect of each compound on the viability of host cells, a CellTiter 96R AQueous One Solution Cell Proliferation assay system reagent was added to the three rows of uninfected HFFs after 72 h of growth. The absorbance was recorded at 490 nm after the addition of reagent for 3 h. The assay was performed in triplicate and repeated three times. Either the 50% toxicity dose (TD_50_) for the host cell, or the 50% inhibitory concentration (IC_50_) for the parasite was calculated, and the therapeutic index (TI) of each compound was defined as TD_50_/IC_50_. A high TI indicates that the compound specifically inhibits *T. gondii* replication and has a negligible effect on HFFs.

### Plaque assay

Plaque analysis was performed as previously described (Liu et al., 2020). In a 24-well plate inoculated with HFF cells, add 5 μM CGI-1746 or JH-II-127 were used to infect HFF with 100 newly released tachyzoites. The parasite treated with DMSO was the negative control, and the 1 μM pyrimethamine group was the positive control. Each treatment was repeated for three times and incubated for 7 days without light. The plates were fixed with methanol and dried overnight at room temperature before staining with 1.5% crystal violet. The 24-well plates were photographed and the plaque area in each well was measured by ImageJ software as a proxy for growth.

### Invasion assay

The invasion assay was conducted according to the experimental conditions previously described (Wilson et al., 2020). The HFF cells infected with GFP-ATG8 (Besteiro et al., 2011) strains were treated with the corresponding concentration of drugs. After 24 hours, the fresh released tachyzoites were added to the plates containing confluent monolayer HFFs on ice for 20min. The plate was further incubated for 30min at 38°C to allow the parasites to invade the host cells. Infected cells were fixed immediately with 4% paraformaldehyde at 37°C for 30 min and treated with murine anti-TgSAG1 polyclonal antibody (1:5000, cat # ab8313; Abcam, USA) incubated for 1 h, then stained with anti-mouse Alexa Fluor 594 (1:1000, Invitrogen, Shanghai, China) for 1 h. Then staining was done with DAPI (4 ‘, 6-diamine-2-phenyllindo) for 5 min. Intracellular parasites are represented by green^+/^red^-^ and extracellular parasites by green^+^/red^+^ (yellow). The ratio of the number of intracellular parasites to the total tachyzoite population is called the invasion ratio. Under fluorescence microscope, 10 fields were randomly selected and counted with 3 replicates.

### Intracellular proliferation assay

Intracellular proliferation tests were performed as described previously (Liu et al., 2020). Fresh tachyzoites of GFP-ATG8 were infected with 24 well plate HFF monolayer cells with the proportion of cells: strain =1:4. Two hours later, the culture medium was replaced with 1% FBS-DMEM culture medium with the corresponding drug concentration. After incubation at 37°C for 24 h, the cells were fixed with pre-cooled methanol for 10min and stained with Reggie’s for 20min. At least 100 parasitophorous vacuole were randomly selected under the oil microscope and the number of *T. gondii* in each parasitophorous vacuole was counted.

### Egress assay

The egress experiment was carried out as previously mentioned (Schultz and Carruthers, 2018) with minor modifications. HFF monolayer in 96-well plates were infected with 5×10^4^ tachyzoites, and then cultured in 1% FBS-DMEM medium containing the corresponding drug at 37°C and 5% CO_2_ for 24 h. Before exergy determination, the Ringer buffer was preheated at 37°C gently washed the plates three times. Then the Zaprinast (Topscience T2129-1mg) was diluted with Ringer buffer to 57 μM, 45 all was added to each well and incubated for 20 min at 37°C with 5%CO_2_. After 20min, the 96-well plates were placed on ice immediately, and the supernatant was transferred into the centrifuge tubes. Centrifuge at 4°C 500 xg for 5 min (pre-cooling of centrifuge in advance), and test directly on Hitachi assembly line.

### Gliding assay

Three vials of HFF cells infected with RH strain were added with 2.5 μM CGI-1746, 2.5 μM JH-II-127 and DMSO, respectively. The fresh parasites were collected and added to the polylysine-coated 24-well plate. Cultured at 37°C for 15 min, fixed with 4% PFA and 0.05% glutaraldehyde for 15 min, and treated with murine anti-TgSAG1 polyclonal antibody (1:5000, Cat # ab8313; Abcam, USA) was incubated for 1 h, and then incubated with anti-mouse Alexa Fluor 594 (1:1000, Invitrogen, Shanghai, China) for 1 h. The parasites were indicated with red^+^, ang sliding trajectory of the tachyzoites was observed under a fluorescence microscope.

### Observation of mitochondria morphology

HFF cells were inoculated in a 24-well plate with glass plates, and each well has 1.4×10^5^ HFF cells infected with the Apicox13 strain, which carries an mNG green fluorescent label to mark mitochondria. CGI-1746, JH-II-127 and DMSO with a concentration of 5 μM were added to the plate, respectively, and cultured for 24h in the dark. Fixed with 4% PFA for 15 min at 37°C, incubated with murine anti-TgSAG1 polyclonal antibody for 1 h, and then stained with anti-mouse Alexa Fluor 594 for 1 h. Then stain with DAPI for 5 min. The cell membrane was represented by red, and the mitochondria was represented by green. The morphology of mitochondria was observed under fluorescence microscope.

### Determination of ATP concentration

ATP concentration determined were performed as described previously (Tjhin et al., 2020). Three vials of HFF cells infected with RH strain were added with 5 μM compounds and DMSO, respectively. The ATP concentration in RH *T. gondii* tachyzoites were measured by the ATP Determination Kit (Cat # A22066, Invitrogen, Shanghai, China). Parasites were harvested from culture bottles, host cell debris was removed through a filter, and fresh parasites were collected after centrifugation at room temperature at 1500×g for 10 min. The parasites were suspended with 500 μL PBS buffer, 100 μL of the suspension was used for protein extraction and the protein concentration was determined by BCA Protein Assay Kit (Beyotime, Shanghai, China), and the remaining 400 μL was used for ATP concentration determination. The supernatant was abandoned by centrifugation, and 100 μL ATP extract was added for re-suspension. The supernatant was collected by centrifugation and bathed in 100°C water for 90 s, and ATP concentration was determined on ice with an ATP assay kit. Parasite extract or ATP standard was added at 10% of the total volume of each reaction, and luminescence was read at 560 nm by microplate reader immediately after the reaction began. The concentration of ATP in the sample is calculated by the standard curve generated by the known concentration of ATP. Finally, the measured protein concentration was normalized. Three independent experiments were evaluated in three replicates.

### Determination of ROS

The intracellular ROS production was measured by Reactive Oxygen Species Assay Kit (Yeasen, Shanghai, China), which is a change in fluorescence intensity based on fluorescent dye DCFH-DA (2, 7 - dichlorodi - hydrocein diacetate). Three vials of HFF cells infected with RH strain were added with 5 μM compounds and DMSO, respectively. Extracellular parasites were collected according to the above methods, washed with PBS buffer solution until the final concentration was 1×10^6^ parasites/ml, and then mixed with 10 μM DCFH-DA. The mixture was incubated at 37°C in the dark for 30min, mixed upside down every 5 min, and detected directly on flow cytometry with 3 replicates.

### Statistical analysis

GraphPad Prism 8.0 software (GraphPad software Inc., San Diego, CA, United States) is used to analyze all data. The data were plotted and represented as three repeated means ± standard deviation (SD). TD_50_ and IC_50_ values were plotted by non-linear regression analysis (curve fitting). Phenotypic analysis data were analyzed by two-tailed Student’s *t*-test or one-way ANOVA combined with Tukey’s *post-hoc* test. *p*<0.05 was considered statistically significant.

## Results

### Preliminary screening small molecule inhibitors of *T. gondii*

**Figure 1:**
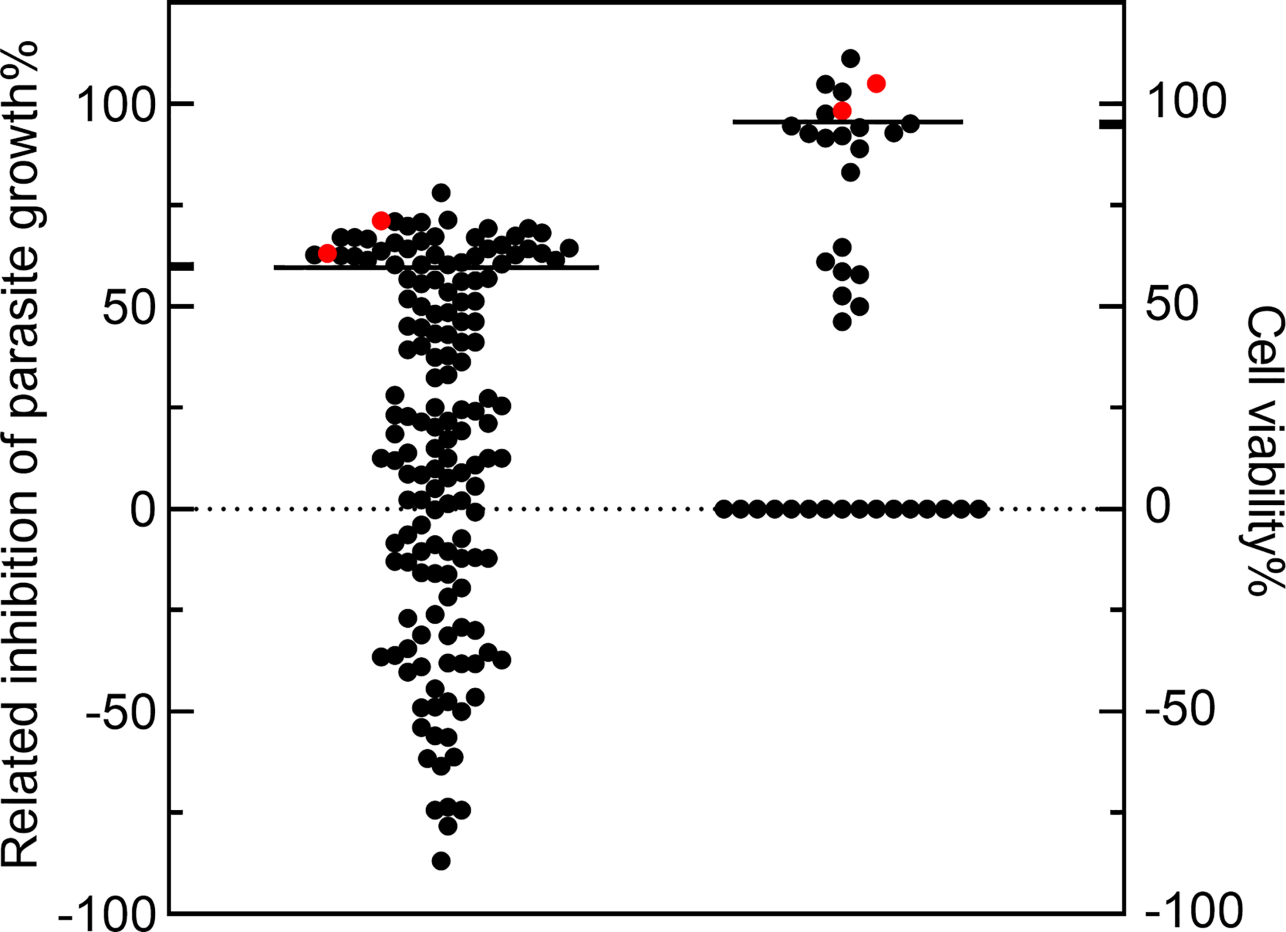
The compounds with high anti-*Toxoplasma gondii* activity and low cytotoxicity were preliminarily screened. The 152 compounds were screened, and the dimethyl sulfoxide (DMSO) group was used as the negative control group to calculate the inhibition rate. Among the 38 compounds, the parasite growth inhibition rate was greater than 60%, and the cell viability of 7 compounds was greater than 95%. CGI-1746 and JH-II-127(red dots) were identified as suitable anti- *T. gondii* drugs.

152 compounds were selected from the National Drug Screening Center of China for preliminary screening of novel drugs against *T. gondii* (**Supplemental Table S1).** Among the 152 compounds, 38 compounds had a parasite growth inhibition rate greater than 60%. Cytotoxicity screening of these 38 compounds showed that 7 compounds had cell viability greater than 95%. Among them, Amiodarone HCl (Bobbala et al., 2010), Loperamide HCl and Loperamide (hydrochloride) (Ch"Ng et al., 2013) had been reported as having anti- parasite effects and were excluded for a further IC_50/_TD_50_ experiment, LRRK2-IN-1 and Dexmedetomidine did not find suitable concentrations to inhibit *T. gondii* but not toxic to cells, so they were excluded. Two compounds were identified, CGI-1746 (parasite inhibition rate: 63.14 ± 0.01%; Host survival rate: 105.15 ± 0.11%) and JH-II-127 (parasite inhibition rate: 71.22 ± 0.00%; Host survival rate: 98.25 ± 0.14%), which meets our criteria for high anti-*T. gondii* activity (≥60% parasite inhibition) and low cytotoxicity (≥95% cell viability) for new clinical anti-*T. gondii* drugs development (**Figure 1**).

### Anti-*T. gondii* activity and cytotoxicity of CGI-1746 and JH-II-127

**Figure 2:**
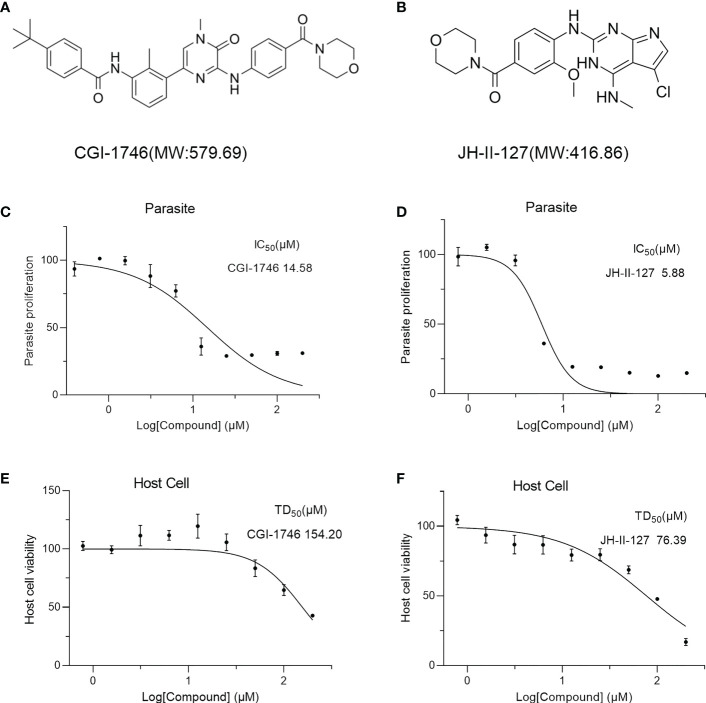
CGI-1746 and JH-II-127 inhibited the growth of *T. gondii* and were effective in preventing toxoplasmosis. **(A, B)** Molecular weight and molecular structure of CGI-1746 and JH-II-127. **(C, D)** Effects of different concentrations of CGI-1746 and JH-II-127 on the growth of *T. gondii*. Different concentrations (0-200 μM) of CGI-1746 and JH-II-127 were incubated with HFFs infected with RH-2F tachyzoites, and β-Gal activity was measured to calculate the IC_50_ value of the parasite. **(E, F)** Effects of different concentrations of CGI-1746 and JH-II-127 on the cytotoxicity of HFF. HFF cells were incubated with different concentrations (0-200 μM) of CGI-1746 and JH-II-127 for 72 h, and TD_50_ values were calculated, respectively. The data of three experiments were mean ± SD.

To further evaluate the anti-*T. gondii* activity of CGI-1746 and JH-II-127. The RH-2F strain expressing β-gal was detected using CGI-1746 and JH-II-127 (**Figures 2A, B**), and colorimetry was allowed to quantify the growth inhibition of *T. gondii*. The results showed that the IC_50_ values of CGI-1746 and JH-II-127 for parasite suppression were 14.58 ± 1.52 and 5.88 ± 0.23 μM, respectively (JH-II-127 with CGI-1746: *p*=0.0006) (**Figures 2C, D**). However, the higher concentrations of those compound keep 30% proliferation, which may be related to cytotoxicity. To analyze the cytotoxicity of each compound to HFF host cells *in vitro*, cell proliferation was measured using MTS assay. TD_50_ values of CGI-1746 and JH-II-127 were 154.20 ± 20.15 and 76.39 ± 14.32 μM, respectively (CGI-1746 with JH-II-127: *p*=0.0055) (**Figures 2E, F**). Based on the above results, the *in vitro* TI of CGI-1746 and JH-II-127 were calculated to be 10.9 and 12.99, respectively.

### Effects of two compounds on intracellular growth of *T. gondii*

**Figure 3:**
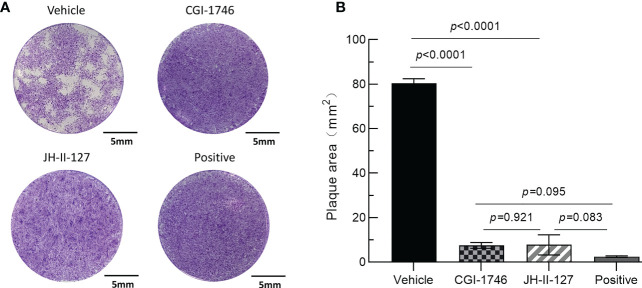
Effects of CGI-1746 and JH-II-127 on the lytic cycle of parasites. **(A)** HFF monolayers infected with 100 tachyzoites were treated with 5 μM CGI-1746, JH-II-127, or pyrimethamine for 7 days. **(B)** The plaque area was measured on the 7th day, and the three experimental data were all mean ± SD, *p*<0.05.

Next, we performed plaque tests to verify the effects of each compound on both parasite and host cells. After 7 days of incubation, we observed that the monolayer of HFF was intact, further indicating that the cytotoxicity of the two compounds was low, but the JH-II-127 treatment may alter cellular morphology compared to positive control and the other compound (**Figure 3A**). Importantly, the parasite patch area with CGI-1746 (7.51 ± 1.29) and JH-II-127 (7.76 ± 4.53) were reduced by approximately 91% and 90%, respectively, compared with the control group (80.44 ± 1.98) (**Figure 3B**). In addition, we noted that the inhibition of CGI-1746 and JH-II-127 on plaque formation was comparable to that of pyrimethamine (2.46 ± 0.36, 97% reduction in plaque area). The results showed that these two compounds indeed inhibit tachyzoite replication.

**Figure 4:**
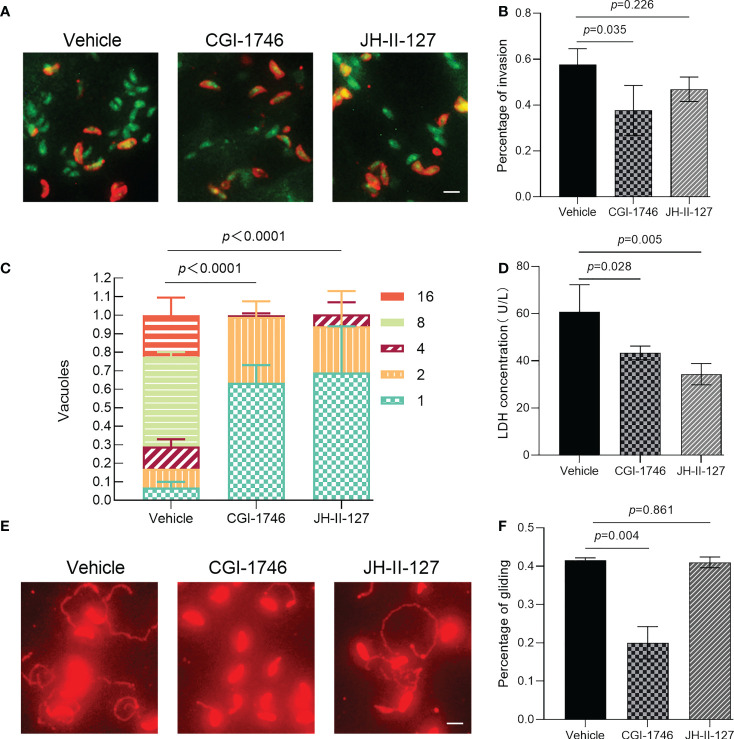
Effects of CGI-1746 and JH-II-127 on the invasion, intracellular proliferation, exocytosis, and gliding ability of *T. gondii*. **(A)** Effects of CGI-1746 and JH-II-127 on the invasivity of *T. gondii*, indicated by green^+/^red^-^ for intracellular parasites and green^+/^red^+^(yellow) for extracellular parasites. **(B)** The number of intracellular parasites to the total number of tachyzoites was calculated as the invasion percentage. **(C)** Effects of CGI-1746 and JH-II-127 on the intracellular proliferation of *T. gondii*. The number of vacuoles containing the different numbers of parasite strains was counted, and the percentage of vacuoles containing different numbers of parasites was expressed as the mean ± SD of the three experimental data. **(D)** Effects of CGI-1746 and JH-II-127 on the exocytosis of *T. gondii*. The parasite was induced by incubation with 57 μM zaprinast for 20 min. The lactate dehydrogenase (LDH) released after induction was used to measure the exocytosis. **(E, F)** Effects of CGI-1746 and JH-II-127 on the gliding ability of *T. gondii*. The slippage of the parasite was observed under a fluorescence microscope, and the slippage ability was calculated as the proportion of the number of parasites with slippage to the total number of tachyzoites. (*p*<0.05, Scale = 5μm, Error bars, SDs of the means from three independent replicates).

The ability of parasite invasion, intracellular proliferation, egress and gliding was observed through parasite phenotype evaluation experiment to determine whether the two compounds affect the specific links in the process of parasite division and proliferation. We found that CGI-1746 can significantly inhibit the invasive ability of parasites, while JH-II-127 has no effect on the invasive ability of parasites, indicated by green^+^/red^-^ for intracellular parasites and green^+^/red^+^(yellow) for extracellular parasites (**Figure 4A**). The number of intracellular parasites to the total number of tachyzoites was calculated as the invasion percentage (CGI-1746: 0.38 ± 0.11; JH-II-127: 0.47 ± 0.05; drug loading control: 0.58 ± 0.07) (**Figure 4B**). In the cell proliferation experiment, these two compounds showed a strong ability to inhibit the growth of tachyzoites in cells. As shown in the figure, most vacuoles have one (DMSO: 0.07 ± 0.04; CGI-1746: 0.64 ± 0.13; JH-II-127: 0.69 ± 0.35), two or four tachyzoites (**Figure 4C**). In contrast, the parasites treated with DMSO were mainly composed of vacuoles containing 8 tachyzoites (DMSO: 0.49 ± 0.04; CGI-1746: 0 ± 0; JH-II-127: 0 ± 0) In the egress experiment, CGI-1746 and JH-II-127 significantly inhibited the egress ability of parasites (CGI-1746: 43.33 ± 2.89; JH-II-127: 34.33 ± 4.51; drug control: 60.67 ± 11.59) (**Figure 4D**). In addition, in the gliding test, CGI-1746 can significantly inhibit the dynamics of parasites, while JH-II-127 does not affect the dynamics of parasites (CGI-1746: 0.20 ± 0.04; JH-II-127: 0.41 ± 0.01; drug loading control: 0.42 ± 0.01) (**Figures 4E, F**).

### Effects of two compounds on mitochondrial

**Figure 5:**
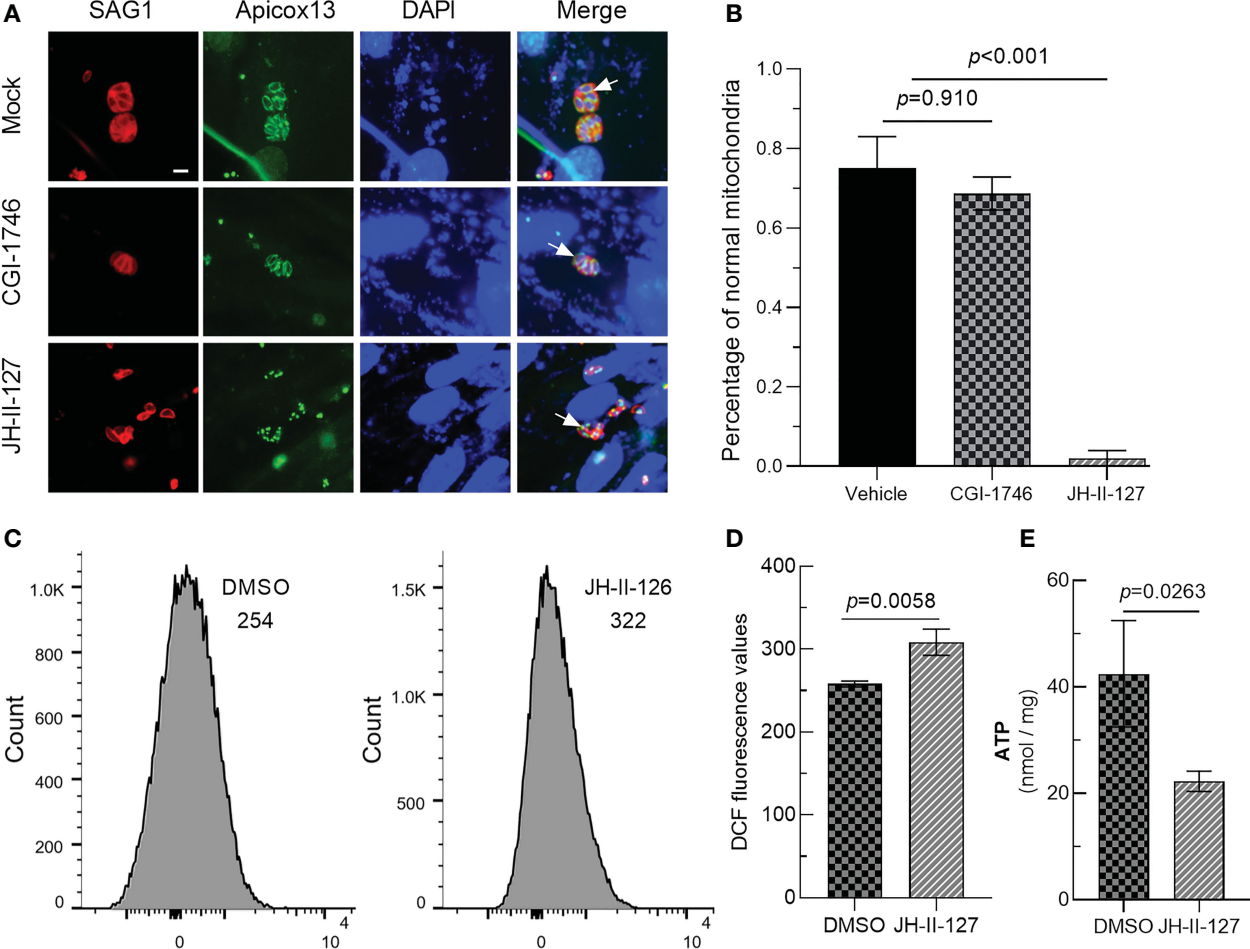
Effects of CGI-1746 and JH-II-127 on mitochondrial of *T. gondii*. **(A)** The mitochondrial morphology of the parasite was observed under the fluorescence microscope. SAG1 indicated the red cell membrane, Apicox13 indicated the green mitochondrial morphology (arrows), and DAPI indicated the blue cell nucleus. **(B)** The proportion of parasites with normal mitochondrial morphology in total tachyzoites was counted. **(C)** DCFH-DA assay was then used to quantify intracellular ROS levels of JH-II-127 on mitochondrial of *T. gondii* **(D)** Fluorescence intensity presented as the mean ± SD error of the mean of three different experiments. **(E)** Whole protein ATP concentration were measured in *T. gondii* of JH-II-127 treatment and DMSO control group. Data show the mean ± SD from three independent experiments (*p*<0.05, Scale = 5μm, Error bars, SDs of the means from three independent replicates).

Mitochondria are important organelles regulating the division cycle of *T. gondii*, so we tried to observe the effects of these two compounds on parasite mitochondria. The IFA results showed that CGI-1746 had no effect on the mitochondria of parasites, but JH-II-127 had an obvious effect on the mitochondria of parasites. The mitochondria of almost all tachyzoites treated with JH-II-127 changed from standard circular or sperm shape to abnormal point shape, where the arrow points (DMSO: 0.75 ± 0.11; CGI-1746: 0.74 ± 0.14; JH-II-127: 0.02 ± 0.02) (**Figures 5A, B**). To investigate whether the effect of JH-II-127 on *T. gondii* is related to oxidative phosphorylation, ROS levels and ATP concentration were measured. After treatment with JH-II-127, fluorescent probe DCFH-DA was used to evaluate the changes of ROS in treatment and control group. Compared with control group, JH-II-127 treatment significantly increased the fluorescence intensity of DCFH-DA (DMSO: 258.00 ± 3.61; JH-II-127: 308.33 ± 15.82) (**Figures 5C, D**). ATP concentration in JH-II-127 treatment and control group were also measured. Compared with control group, ATP concentration of *T. gondii* was significantly decreased after JH-II-127 treatment (DMSO: 42.49 ± 10.00; JH-II-127: 22.28 ± 1.90) (**Figure 5E**).

## Discussion

In this study, 152 compounds associated with autophagy pathway were screened and evaluated to reuse as potently clinical drugs, which can inhibit *T. gondii* growth and have no host cytotoxicity. Our results indicate that CGI-1746 and JH-II-127 have high anti-*T. gondii* activity and low cytotoxicity. In addition, inhibited parasite growth compounds will maximize host cell selectivity. Therefore, the selectivity of these two compounds was evaluated by the therapeutic index. We found that the selectivity of CGI-1746 and JH-II-127 against *T. gondii* on host cells increased the effectiveness by about 10 times. Importantly, plaque tests showed that the inhibitory activity of these two compounds was comparable to that of pyrimethamine (**Figure 3B**). A previous investigation also showed that the TI values of sulfadiazine and pyrimethamine currently used in the clinical treatment of toxoplasmosis were ≤1 and ≤8 respectively (Kamau et al., 2012). Taken together, these findings suggest that CGI-1746 and JH-II-127 could be potentially repurposed as candidate drugs against *T. gondii* infection.

Bruton’s tyrosine kinase (Btk) is a therapeutic target for malignant B-cell tumors and immune system diseases (Tsukada et al., 1994; Satterthwaite and Witte, 2000). CGI-1746 is a small-molecule inhibitor that effectively inhibits the activity of Btk by inhibiting the autophosphorylation and transphosphorylation necessary for Btk activation. It is also the first compound reported to bind to a BTK-specific inactive conformation to produce excellent kinase selectivity (Di Paolo et al., 2011). In cell experiments, the compound blocked BCR-mediated b-cell proliferation and inhibited the production of TNF-α, IL-1β and IL-6 induced by Fc-γRIII in macrophages. In experimental mouse models, CGI-1746 showed strong antiarthritic activity, manifested by reduced levels of cytokines and autoantibodies in the joints (Di Paolo et al., 2011).

*T. gondii* and other parietal parasitoids must rely on their movement in invading host cells. However, due to the lack of motor organelles such as pseudopodia, cilia and flagella, they must rely on actomyosin motor (AMM) device provides power (Baum et al., 2006), and a variety of proteins secreted by secretory organelles such as microwires, rod-shaped bodies and dense particles participate in the completion (Sharma and Chitnis, 2013). It has also been previously reported that anti-*T. gondii* drug targets involved in parasite motility and host-cell invasion (Silva et al., 2021).In our phenotypic experiments, CGI-1746 was found to significantly inhibit parasite invasion, egress, intracellular proliferation and gliding abilities, but did not affect mitochondrial morphological structure. These results suggest that CGI-1746 inhibited the division and proliferation of *T. gondii* tachyzoites mainly by affecting the function and gliding ability of tachyzoite secretory organelles of *T. gondii*.

Related studies have shown that LRRK2 (Leucine-Rich Repeat Kinase 2) is a potential improvement and therapeutic target for Parkinson’s disease. Inhibition of LRRK2 activity and the resulting improvement of membrane transport and lysosome function is a promising new treatment for Parkinson’s disease (Jennings et al., 2022). It has been found that JH-II-127(2-anilino-4-methylamino-5-chloropyrrolopyrimidine) is a highly effective inhibitor of selective LRRK2 kinase activity. The activity of wild-type LRRK2 and LRRK2- G2019S mutant was inhibited by significantly inhibiting phosphorylation at Ser910 and Ser935 (Hatcher et al., 2015).

In *T. gondii*, mitochondria are important organelles regulating the division cycle of *T. gondii*, and many proteins located in mitochondria are involved in the regulation of the division cycle of *T. gondii (* Radke et al., 2001; Ouologuem and Roos, 2014). It has also been previously reported that anti-*T. gondii* drug targets involved in mitochondrial electron transport pathway (Alday and Doggett, 2017). Atovaquone is a broad-spectrum antiparasitic drug that selectively inhibits mitochondrial electron transport in the cytochrome *bc*_1_ complex and disrupts mitochondrial membrane potential (Srivastava and Vaidya, 1999). *T. gondii* and malaria-related *Plasmodium falciparum* could both killed specifically by the mitochondrial inhibitor atovaquone (Mather et al., 2007). In this paper, we show that JH-II-127 could significantly inhibit the egress and intracellular proliferation of the parasite, but it was more noteworthy that JH-II-127 had a significant effect on the parasite mitochondria, and almost all mitochondria treated by JH-II-127 transformed from normal ring or spermatozoa into abnormal point shape. Therefore, we tentatively put forward that JH-II-127 inhibits the proliferation of *T. gondii* mainly by destroying the morphology of mitochondria. Further experiments confirmed that the mitochondrial ATP concentration of *T. gondii* decreased and ROS levels increased. Therefore, the JH-II-127 effect of anti- *T. gondii* activity may be related to the damage of the mitochondrial electron transport chain, but it needs to be confirmed by further studies.

Although both CGI-1746 and JH-II-127 showed excellent anti-parasite activity, showing the good inhibitory effect on parasite growth and low cytotoxicity to host cells, further determination of treatment concentration and duration is needed to evaluate the efficacy of these two compounds in *T. gondii* infected mice. At the same time, due to the lack of Toxoplasma type II strains, we were unable to assess whether these two compounds inhibit the formation of cysts or eliminate bradyzoites. The above experimental results suggest that the inhibitory effect of CGI-1746 is related to the function of secretory organelles as well as gliding ability, and the inhibitory effect of JH-II-127 is related to the damage of mitochondrial electron transport chain, and both of these compounds are inhibitors against autophagy pathway, but the corresponding targets of these two compounds in *T. gondii* have not been identified; therefore, the exact mechanism is unclear and needs to be investigated in the next step.

In brief, we identified two potential drugs with high anti-*T. gondii* activity and low cytotoxicity from 152 autophagy pathway related small molecule inhibitors from National Drug Screening Center of China. Although the mechanism of action against *T. gondii* infection requires further investigation, the available data suggest that these two compounds hold promise as drug candidates for the treatment of toxoplasmosis. This paper provides a possibility for further screening of antiparasitic drugs for drug repurposing and provides a new idea for further research on small-molecule compounds as effective antiparasitic drugs.

## Data availability statement

The original contributions presented in the study are included in the article/**Supplementary Material**. Further inquiries can be directed to the corresponding authors.

## Author contributions

FT, Y-NM and Q-QH conceived and designed the study. Q-QH, X-JL, S-PX, L-YJ, J-HC and J-MS performed experiments. Y-NM and Q-QH analyzed the data. Y-NM and FT prepared figures and tables and wrote the manuscript with input from all authors. All the authors read and approved the final manuscript.

## Supplementary material

Supplementary Table 1 The 152 compounds’ parasite inhibition (%) and host viability (%).
