# Corrigendum: Two small-molecule inhibitors of *Toxoplasma gondii* proliferation *in vitro*

**DOI:** 10.3389/fcimb.2023.1332786

**Published:** 2023-12-01

**Authors:** Qian-qian Hua, Xue-jing Lin, Shi-peng Xiang, Li-ya Jiang, Jin-hao Cai, Jian-min Sun, Feng Tan, Ya-ni Mou

**Affiliations:** ^1^Department of Clinical Laboratory, Affiliated Dongyang Hospital of Wenzhou Medical University, Dongyang, Zhejiang, China; ^2^Department of Parasitology, School of Basic Medical Sciences, Wenzhou Medical University, Wenzhou, Zhejiang, China

**Keywords:** CGI-1746, JH-II-127, *Toxoplasma gondii*, infection, *in vitro*

## Affiliations

In the published article, there was an error regarding affiliation 1. Affiliation 1 was published as: Clinical Laboratory, Dongyang People’s Hospital, Jinhua, Zhejiang, China. The correct affiliation is: Department of Clinical Laboratory, Affiliated Dongyang Hospital of Wenzhou Medical University, Dongyang, Zhejiang, China.

The authors apologize for this error and state that this does not change the scientific conclusions of the article in any way. The original article has been updated.

